# Dichlorido(6-methyl-2,2′-bipyridine-κ^2^ *N*,*N*′)zinc(II)

**DOI:** 10.1107/S1600536808028894

**Published:** 2008-09-13

**Authors:** Roya Ahmadi, Khadijeh Kalateh, Amin Ebadi, Vahid Amani, Hamid Reza Khavasi

**Affiliations:** a Islamic Azad University, Shahr-e-Rey Branch, Tehran, Iran; b Department of Chemistry, Islamic Azad University, Kazeroon Branch, Kazeroon, Fars, Iran; c Department of Chemistry, Shahid Beheshti University, Tehran 1983963113, Iran

## Abstract

In the mol­ecule of the title compound, [ZnCl_2_(C_11_H_10_N_2_)], the Zn^II^ atom is four-coordinated in a distorted tetra­hedral configuration by two N atoms from the 6-methyl-2,2′-bipyridine ligand and by two Cl atoms. There are π–π contacts between the pyridine ring and the five-membered ring, and also between the pyridine rings, [centroid–centroid distances = 3.685 (3) and 3.757 (3) Å, respectively].

## Related literature

For related literature, see: Ahmadi *et al.* (2008 ▶); Yousefi *et al.* (2008 ▶); Khan & Tuck (1984 ▶); Gruia *et al.* (2007 ▶); Kozhevnikov *et al.* (2006 ▶); Reimann *et al.* (1966 ▶); Preston & Kennard (1969 ▶); Liu *et al.* (2004 ▶); Khavasi *et al.* (2008 ▶); Khalighi *et al.* (2008 ▶); Steffen & Palenik (1976 ▶, 1977 ▶); Qin *et al.* (1999 ▶); Lundberg (1966 ▶).
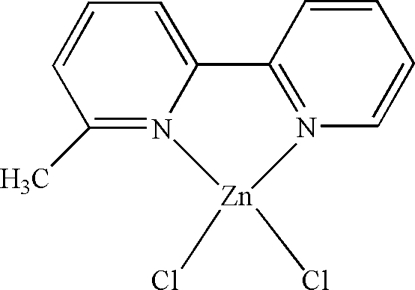


## Experimental

### 

#### Crystal data

[ZnCl_2_(C_11_H_10_N_2_)]
*M* *_r_* = 306.50
Monoclinic, 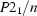

*a* = 7.4674 (15) Å
*b* = 9.5105 (17) Å
*c* = 17.656 (4) Å
β = 96.551 (18)°
*V* = 1245.7 (4) Å^3^
*Z* = 4
Mo *K*α radiation
μ = 2.37 mm^−1^
*T* = 298 (2) K
0.30 × 0.15 × 0.10 mm

#### Data collection

Bruker SMART CCD area-detector diffractometer
Absorption correction: multi-scan (*SADABS*; Sheldrick, 1998 ▶) *T*_min_ = 0.668, *T*_max_ = 0.802
10401 measured reflections
3358 independent reflections
2576 reflections with *I* > 2σ(*I*)
*R*_int_ = 0.078

#### Refinement

*R*[*F* ^2^ > 2σ(*F* ^2^)] = 0.060
*wR*(*F* ^2^) = 0.130
*S* = 1.17
3358 reflections
145 parameters
H-atom parameters constrained
Δρ_max_ = 1.04 e Å^−3^
Δρ_min_ = −0.70 e Å^−3^

### 

Data collection: *SMART* (Bruker, 1998 ▶); cell refinement: *SAINT* (Bruker, 1998 ▶); data reduction: *SAINT*; program(s) used to solve structure: *SHELXTL* (Sheldrick, 2008 ▶); program(s) used to refine structure: *SHELXTL*; molecular graphics: *ORTEP-3 for Windows* (Farrugia, 1997 ▶); software used to prepare material for publication: *WinGX* (Farrugia, 1999 ▶).

## Supplementary Material

Crystal structure: contains datablocks I. DOI: 10.1107/S1600536808028894/hk2529sup1.cif

Structure factors: contains datablocks I. DOI: 10.1107/S1600536808028894/hk2529Isup2.hkl

Additional supplementary materials:  crystallographic information; 3D view; checkCIF report

## Figures and Tables

**Table:** 

Zn1—N1	2.066 (4)
Zn1—N2	2.053 (4)
Zn1—Cl1	2.2236 (15)
Zn1—Cl2	2.1995 (13)

**Table:** 

N1—Zn1—Cl1	111.08 (11)
N2—Zn1—Cl1	109.16 (11)
Cl2—Zn1—Cl1	116.72 (5)
N1—Zn1—Cl2	116.84 (11)
N2—Zn1—Cl2	117.28 (10)
N2—Zn1—N1	80.31 (15)

**Fig. 1.**
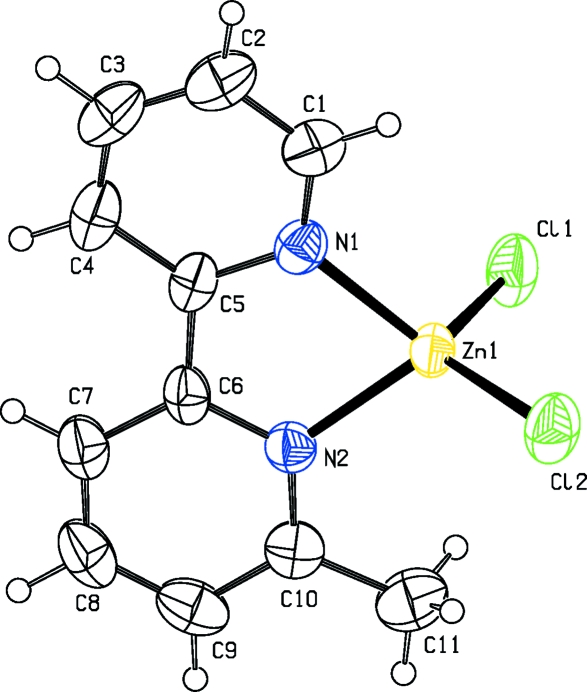
The molecular structure of the title molecule, with the atom-numbering scheme. Displacement ellipsoids are drawn at the 40% probability level.

**Fig. 2.**
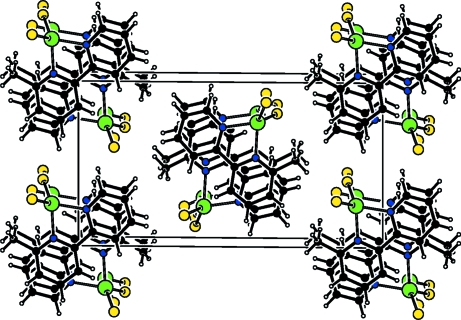
A packing diagram of the title compound.

## supplementary crystallographic information

### Comment

Recently, we reported the syntheses and crystal structures of [Cd(5,5'-dmbpy)(µ-Cl)_2_]_n_, (II), (Ahmadi *et al.*,  2008) and [Hg(4,4'-dmbpy)I_2_], (III), (Yousefi *et al.*,  2008) [where 5,5'-dmbpy is 5,5'-dimethyl-2,2'-bipyridine and 4,4'-dmbpy is 4,4'-dimethyl-2,2'-bi- pyridine]. There are several Zn^II^ complexes, with formula, [ZnCl_2_(N-N)], such as [ZnCl_2_(bipy)], (IV), (Khan & Tuck, 1984), [ZnCl_2_(biim)], (V), (Gruia *et al.*,  2007), [ZnCl_2_(phbipy)], (VI), (Kozhevnikov *et al.*,  2006), [ZnCl_2_(phen)], (VII), (Reimann *et al.*,  1966), [ZnCl_2_(dmphen)], (VIII), (Preston & Kennard, 1969), [ZnCl_2_(dpdmbip)], (IX), (Liu *et al.*,  2004), [ZnCl_2_(dm4bt)], (X), (Khavasi *et al.*,  2008) and [Zn(5,5'-dmbpy)Cl_2_], (XI), (Khalighi *et al.*,  2008) [where bipy is 2,2'-bipyridine, biim is 2,2'-biimidazole, phbipy is 5-phenyl-2,2'-bipyridine, phen is 1,10-phenanthroline, dmphen is 2,9-dimethyl-1,10-phenanthroline, dpdmbip is 4,4'-diphenyl-6,6'-dimethyl-2,2'-bipyrimidine and dm4bt is 2,2'-dimethyl-4,4'-bithiazole] have been synthesized and characterized by single-crystal X-ray diffraction methods.

There are several Zn^II^ complexes, with formula, [ZnCl_2_*L*_2_], such as [ZnCl_2_(py)_2_], (XII), (Steffen & Palenik, 1976), [ZnCl_2_(4-cypy)_2_], (XIII), (Steffen & Palenik, 1977), [ZnCl_2_(2-ampy)_2_], (XIV), (Qin *et al.*,  1999) and [ZnCl_2_(im)_2_], (XV), (Lundberg, 1966) [where py is pyridine, 4-cypy is 4-cyanopyridine, 2-ampy is 2-aminopyridine and im is imidazole] have been synthesized and characterized by single-crystal X-ray diffraction methods. We report herein the synthesis and crystal structure of the title compound, (I).

In the title compound, (I), (Fig. 1), the Zn^II^ atom is four-coordinated in a distorted tetrahedral configuration by two N atoms from 6-methyl-2,2'-bi- pyridine and two Cl atoms. The Zn-Cl and Zn-N bond lengths and angles (Table 1) are within normal ranges, as in (IV), (VII), (X) and (XI).

In the crystal structure, the π—π contacts (Fig. 2) between the rings A (Zn1/N1/N2/C5/C6) and C (N2/C6-C10), and also between the pyridine rings B (N1/C1-C5) and C, Cg1···Cg3^i^ and Cg2···Cg3^ii^ [symmetry codes: (i) -x, -y, -z; (ii) 1 - x, -y, -z, where Cg1, Cg2 and Cg3 are the centroids of the rings A (Zn1/N1/N2/C5/C6), B (N1/C1-C5) and C (N2/C6-C10), respectively] may stabilize the structure, with centroid-centroid distances of 3.685 (3) Å and 3.757 (3) Å, respectively.

### Experimental

For the preparation of the title compound, (I), a solution of 6-methyl-2,2' -bipyridine (0.15 g, 0.88 mmol) in methanol (10 ml) was added to a solution of ZnCl_2_ (0.12 g, 0.88 mmol) in acetonitrile (30 ml) and the resulting colorless solution was stirred for 20 min at at 313 K, and then it was left to evaporate slowly at room temperature. After one week, colorless block crystals of the title compound were isolated (yield; 0.19 g, 70.4%).

### Refinement

H atoms were positioned geometrically, with C-H = 0.93 and 0.96 Å for aromatic and methyl H, respectively, and constrained to ride on their parent atoms with U_iso_(H) = 1.2U_eq_(C).

### Crystal data

**Table:**

[ZnCl_2_(C_11_H_10_N_2_)]	*F*(000) = 616
*M**_r_* = 306.50	*D*_x_ = 1.634 Mg m^−^^3^
Monoclinic, *P*2_1_/*n*	Mo *K*α radiation, λ = 0.71073 Å
Hall symbol: -P 2yn	Cell parameters from 1987 reflections
*a* = 7.4674 (15) Å	θ = 2.3–29.2°
*b* = 9.5105 (17) Å	µ = 2.37 mm^−^^1^
*c* = 17.656 (4) Å	*T* = 298 K
β = 96.551 (18)°	Block, colorless
*V* = 1245.7 (4) Å^3^	0.30 × 0.15 × 0.10 mm
*Z* = 4	

### Data collection

**Table:**

Bruker SMART CCD area-detector diffractometer	3358 independent reflections
Radiation source: fine-focus sealed tube	2576 reflections with *I* > 2σ(*I*)
graphite	*R*_int_ = 0.078
φ and ω scans	θ_max_ = 29.2°, θ_min_ = 2.3°
Absorption correction: multi-scan (SADABS; Sheldrick, 1998)	*h* = −10→10
*T*_min_ = 0.668, *T*_max_ = 0.802	*k* = −12→13
10401 measured reflections	*l* = −24→24

### Refinement

**Table:**

Refinement on *F*^2^	Primary atom site location: structure-invariant direct methods
Least-squares matrix: full	Secondary atom site location: difference Fourier map
*R*[*F*^2^ > 2σ(*F*^2^)] = 0.060	Hydrogen site location: inferred from neighbouring sites
*wR*(*F*^2^) = 0.130	H-atom parameters constrained
*S* = 1.17	*w* = 1/[σ^2^(*F*_o_^2^) + (0.0315*P*)^2^ + 1.9318*P*] where *P* = (*F*_o_^2^ + 2*F*_c_^2^)/3
3358 reflections	(Δ/σ)_max_ = 0.004
145 parameters	Δρ_max_ = 1.05 e Å^−^^3^
0 restraints	Δρ_min_ = −0.70 e Å^−^^3^

### Special details

**Table:**

Geometry. All e.s.d.'s (except the e.s.d. in the dihedral angle between two l.s. planes) are estimated using the full covariance matrix. The cell e.s.d.'s are taken into account individually in the estimation of e.s.d.'s in distances, angles and torsion angles; correlations between e.s.d.'s in cell parameters are only used when they are defined by crystal symmetry. An approximate (isotropic) treatment of cell e.s.d.'s is used for estimating e.s.d.'s involving l.s. planes.
Refinement. Refinement of *F*^2^ against ALL reflections. The weighted *R*-factor *wR* and goodness of fit *S* are based on *F*^2^, conventional *R*-factors *R* are based on *F*, with *F* set to zero for negative *F*^2^. The threshold expression of *F*^2^ > σ(*F*^2^) is used only for calculating *R*-factors(gt) *etc*. and is not relevant to the choice of reflections for refinement. *R*-factors based on *F*^2^ are statistically about twice as large as those based on *F*, and *R*- factors based on ALL data will be even larger.

### Fractional atomic coordinates and isotropic or equivalent isotropic displacement parameters (Å^2^)

**Table:**

	*x*	*y*	*z*	*U*_iso_*/*U*_eq_	
Zn1	0.26730 (7)	0.26879 (5)	0.08829 (3)	0.04730 (16)	
Cl1	0.53626 (17)	0.30874 (16)	0.15251 (8)	0.0708 (4)	
Cl2	0.04386 (18)	0.40228 (15)	0.11668 (9)	0.0701 (4)	
N1	0.2881 (5)	0.2367 (4)	−0.0261 (2)	0.0494 (8)	
N2	0.2225 (5)	0.0559 (4)	0.0820 (2)	0.0460 (8)	
C1	0.3141 (7)	0.3363 (6)	−0.0782 (3)	0.0619 (12)	
H1	0.3145	0.4304	−0.0639	0.074*	
C2	0.3400 (8)	0.3028 (7)	−0.1517 (3)	0.0741 (16)	
H2	0.3573	0.3733	−0.1867	0.089*	
C3	0.3401 (8)	0.1653 (7)	−0.1730 (3)	0.0726 (16)	
H3	0.3590	0.1414	−0.2226	0.087*	
C4	0.3121 (6)	0.0608 (6)	−0.1210 (3)	0.0626 (13)	
H4	0.3120	−0.0335	−0.1350	0.075*	
C5	0.2840 (5)	0.1004 (5)	−0.0466 (2)	0.0465 (9)	
C6	0.2434 (5)	−0.0003 (5)	0.0127 (3)	0.0469 (9)	
C7	0.2248 (7)	−0.1435 (5)	0.0005 (3)	0.0606 (12)	
H7	0.2412	−0.1824	−0.0465	0.073*	
C8	0.1819 (7)	−0.2268 (6)	0.0589 (4)	0.0698 (15)	
H8	0.1668	−0.3230	0.0512	0.084*	
C9	0.1608 (7)	−0.1700 (6)	0.1293 (4)	0.0679 (14)	
H9	0.1323	−0.2268	0.1692	0.082*	
C10	0.1834 (6)	−0.0254 (5)	0.1392 (3)	0.0566 (11)	
C11	0.1652 (11)	0.0450 (7)	0.2132 (3)	0.091 (2)	
H11A	0.0699	0.1132	0.2063	0.109*	
H11B	0.2763	0.0912	0.2311	0.109*	
H11C	0.1376	−0.0239	0.2498	0.109*	

### Atomic displacement parameters (Å^2^)

**Table:**

	*U*^11^	*U*^22^	*U*^33^	*U*^12^	*U*^13^	*U*^23^
Zn1	0.0498 (3)	0.0446 (3)	0.0483 (3)	−0.0009 (2)	0.0092 (2)	−0.0117 (2)
Cl1	0.0512 (6)	0.0826 (9)	0.0770 (9)	0.0013 (6)	0.0008 (6)	−0.0358 (7)
Cl2	0.0599 (7)	0.0712 (8)	0.0801 (9)	0.0137 (6)	0.0111 (6)	−0.0207 (7)
N1	0.0449 (18)	0.056 (2)	0.0475 (19)	0.0037 (16)	0.0055 (15)	−0.0037 (17)
N2	0.0411 (18)	0.0465 (18)	0.050 (2)	−0.0016 (14)	0.0053 (15)	−0.0024 (15)
C1	0.060 (3)	0.068 (3)	0.059 (3)	0.005 (2)	0.012 (2)	0.007 (2)
C2	0.078 (4)	0.096 (5)	0.051 (3)	0.004 (3)	0.015 (3)	0.010 (3)
C3	0.068 (3)	0.107 (5)	0.044 (3)	0.010 (3)	0.011 (2)	−0.004 (3)
C4	0.052 (3)	0.077 (3)	0.056 (3)	0.014 (2)	−0.001 (2)	−0.023 (3)
C5	0.0370 (19)	0.058 (2)	0.043 (2)	0.0091 (18)	−0.0018 (16)	−0.0112 (18)
C6	0.0346 (19)	0.048 (2)	0.056 (2)	0.0026 (17)	−0.0032 (17)	−0.0144 (19)
C7	0.057 (3)	0.049 (3)	0.074 (3)	0.001 (2)	0.004 (2)	−0.014 (2)
C8	0.058 (3)	0.043 (2)	0.106 (4)	0.000 (2)	0.001 (3)	−0.009 (3)
C9	0.057 (3)	0.058 (3)	0.088 (4)	−0.003 (2)	0.003 (3)	0.022 (3)
C10	0.050 (2)	0.060 (3)	0.059 (3)	0.002 (2)	0.003 (2)	0.002 (2)
C11	0.129 (6)	0.093 (5)	0.053 (3)	−0.010 (4)	0.025 (4)	0.013 (3)

### Geometric parameters (Å, °)

**Table:**

Zn1—N1	2.066 (4)	C5—C6	1.475 (7)
Zn1—N2	2.053 (4)	C6—N2	1.360 (5)
Zn1—Cl1	2.2236 (15)	C6—C7	1.384 (6)
Zn1—Cl2	2.1995 (13)	C7—C8	1.367 (8)
C1—N1	1.350 (6)	C7—H7	0.9300
C1—C2	1.371 (7)	C8—C9	1.381 (8)
C1—H1	0.9300	C8—H8	0.9300
C2—C3	1.361 (9)	C9—C10	1.394 (7)
C2—H2	0.9300	C9—H9	0.9300
C3—C4	1.385 (8)	C10—N2	1.329 (6)
C3—H3	0.9300	C10—C11	1.488 (8)
C4—C5	1.406 (6)	C11—H11A	0.9600
C4—H4	0.9300	C11—H11B	0.9600
C5—N1	1.345 (6)	C11—H11C	0.9600
			
N1—Zn1—Cl1	111.08 (11)	N1—C5—C4	120.5 (5)
N2—Zn1—Cl1	109.16 (11)	N1—C5—C6	115.8 (4)
Cl2—Zn1—Cl1	116.72 (5)	C4—C5—C6	123.7 (4)
N1—Zn1—Cl2	116.84 (11)	N2—C6—C7	120.5 (5)
N2—Zn1—Cl2	117.28 (10)	N2—C6—C5	115.9 (4)
N2—Zn1—N1	80.31 (15)	C7—C6—C5	123.6 (4)
C1—N1—Zn1	126.6 (3)	C8—C7—C6	118.7 (5)
C5—N1—Zn1	113.8 (3)	C8—C7—H7	120.6
C5—N1—C1	119.6 (4)	C6—C7—H7	120.6
C6—N2—Zn1	113.6 (3)	C7—C8—C9	120.9 (5)
C10—N2—Zn1	125.5 (3)	C7—C8—H8	119.5
C10—N2—C6	120.8 (4)	C9—C8—H8	119.5
N1—C1—C2	121.9 (5)	C8—C9—C10	118.2 (5)
N1—C1—H1	119.0	C8—C9—H9	120.9
C2—C1—H1	119.0	C10—C9—H9	120.9
C3—C2—C1	119.3 (5)	N2—C10—C9	120.9 (5)
C3—C2—H2	120.3	N2—C10—C11	117.1 (5)
C1—C2—H2	120.3	C9—C10—C11	122.1 (5)
C2—C3—C4	120.1 (5)	C10—C11—H11A	109.5
C2—C3—H3	120.0	C10—C11—H11B	109.5
C4—C3—H3	120.0	H11A—C11—H11B	109.5
C3—C4—C5	118.5 (5)	C10—C11—H11C	109.5
C3—C4—H4	120.7	H11A—C11—H11C	109.5
C5—C4—H4	120.7	H11B—C11—H11C	109.5
			
N2—Zn1—N1—C5	−6.9 (3)	C6—C5—N1—Zn1	7.2 (5)
Cl2—Zn1—N1—C5	−122.7 (3)	C6—C5—N1—C1	−176.3 (4)
Cl1—Zn1—N1—C5	100.0 (3)	N1—C5—C6—N2	−2.7 (5)
N2—Zn1—N1—C1	176.9 (4)	C4—C5—C6—N2	179.1 (4)
Cl2—Zn1—N1—C1	61.1 (4)	N1—C5—C6—C7	176.6 (4)
Cl1—Zn1—N1—C1	−76.2 (4)	C4—C5—C6—C7	−1.6 (7)
Cl1—Zn1—N2—C10	73.8 (4)	C5—C6—N2—Zn1	−3.3 (4)
Cl2—Zn1—N2—C10	−61.8 (4)	C5—C6—N2—C10	179.1 (4)
N1—Zn1—N2—C10	−177.1 (4)	C7—C6—N2—Zn1	177.4 (3)
Cl1—Zn1—N2—C6	−103.7 (3)	C7—C6—N2—C10	−0.2 (6)
Cl2—Zn1—N2—C6	120.7 (3)	N2—C6—C7—C8	1.2 (7)
N1—Zn1—N2—C6	5.4 (3)	C5—C6—C7—C8	−178.1 (4)
C2—C1—N1—C5	−1.2 (7)	C6—C7—C8—C9	−1.2 (8)
C2—C1—N1—Zn1	174.8 (4)	C7—C8—C9—C10	0.3 (8)
N1—C1—C2—C3	−0.2 (9)	C8—C9—C10—N2	0.7 (8)
C1—C2—C3—C4	0.8 (9)	C8—C9—C10—C11	−179.4 (6)
C2—C3—C4—C5	0.0 (8)	C9—C10—N2—Zn1	−178.0 (4)
C3—C4—C5—N1	−1.4 (7)	C9—C10—N2—C6	−0.7 (7)
C3—C4—C5—C6	176.7 (4)	C11—C10—N2—Zn1	2.0 (6)
C4—C5—N1—Zn1	−174.5 (3)	C11—C10—N2—C6	179.3 (5)
C4—C5—N1—C1	2.0 (6)		
